# Familial Hemiplegic Migraine Type 3 (FHM3) With an *SCN1A* Mutation in a Chinese Family: A Case Report

**DOI:** 10.3389/fneur.2018.00976

**Published:** 2018-11-15

**Authors:** Na Shao, Haining Zhang, Xue Wang, Wuqiong Zhang, Miaomiao Yu, Hongmei Meng

**Affiliations:** Department of Neurology and Neuroscience Center, First Hospital of Jilin University, Changchun, China

**Keywords:** migraine, auras, transient ischemic attack, *SCN1A* mutation, familial hemiplegic migraine type 3

## Abstract

Familial hemiplegic migraine (FHM) is a rare, monogenic, autosomal dominant subtype of migraine, in which three genes, *CACNA1A, ATP1A2*, and *SCN1A*, are currently known to be involved. The familial hemiplegic migraine type 3 (FHM3) is seldom caused by mutations in *SCN1A*. Here, we report a rare case of an *SCN1A* mutation leading to FHM3 in a Chinese family. This case report describes a 62-year-old female patient that was admitted to our clinic. She presented with recurrent attacks of hemiplegic migraine. Her symptoms were first suspicious of a transient ischemic attack (TIA), but they were eventually diagnosed as FHM with a c.4495T>C mutation being found in the *SCN1A* gene. This case highlights that when a patient presents at the clinic with TIA symptoms associated with migraine, the diagnosis of an FHM should be considered and a genetic test is indicated. The identification of *SCN1A* gene mutations may help us to further understand the FHM pathophysiology.

## Introduction

Familial hemiplegic migraine (FHM) is a rare autosomal-dominant disease with aura and characterized by transient hemiparesis of some degree that may begin at any age (1). FHMs as a monogenic subtype of migraine are associated with several gene mutations in which three genes, *CACNA1A, ATP1A2*, and *SCN1A*, are currently known to be involved (2–4). In contrast to migraine, a frequent paroxysmal neurological disease, FHM is rarely seen in a clinical setting. Each type of FHM is associated with mutations in a representative gene. The neuronal calcium channel gene *CACNA1A* was the first identified gene and is most frequently affected in FHM1 families. *ATP1A2*, which encodes the α2 subunit of a Na^+^/K^+^-ATPase, is involved in approximately 20% of FHM2 families (5). Mutations causing FHM3 have been identified in the *SCN1A* gene, which encodes the neuronal voltage-gated Na^+^ channel subunit Na_v_1.1 (3, 6). FHM caused by *CAHNA1A or ATP1A2* mutations have been reported in many studies, while *SCN1A* gene-related familial hemiplegic migraine type 3 (FHM3) is less often observed. With over 150 reported mutations, the *SCN1A* gene has frequently been linked to epilepsy (3). However, only ten missense mutations of the *SCN1A* gene were reported in the literature to cause FHM3 (6), of which the mutation (c.4495T>C) has only been detected in a single Swiss family (5). In the present study, we describe a case of FHM3 caused by the mutation (c.4495T>C) of the *SCN1A* gene in a Chinese family.

## Case report

**Figure 1:**
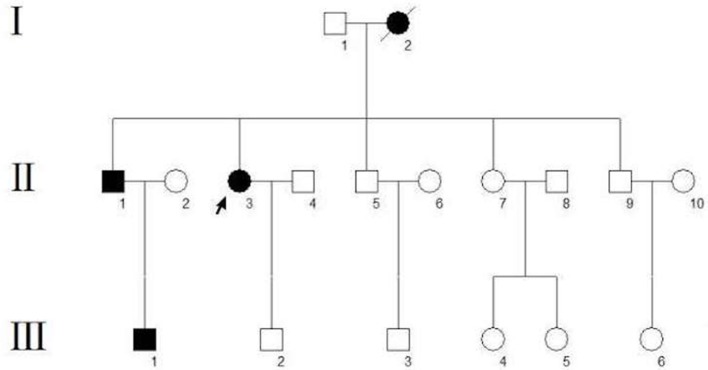
Pedigree of the family. The arrow indicates the proband. Circles indicate females, squares indicate males. The diagonal line indicates a deceased family member. Black squares/circles indicate a carrier of the FHM3 (c.4495T>C) mutation with hemiplegic migraine. White squares/circles indicate subjects that are neither patients nor mutation carriers.

**Figure 2:**
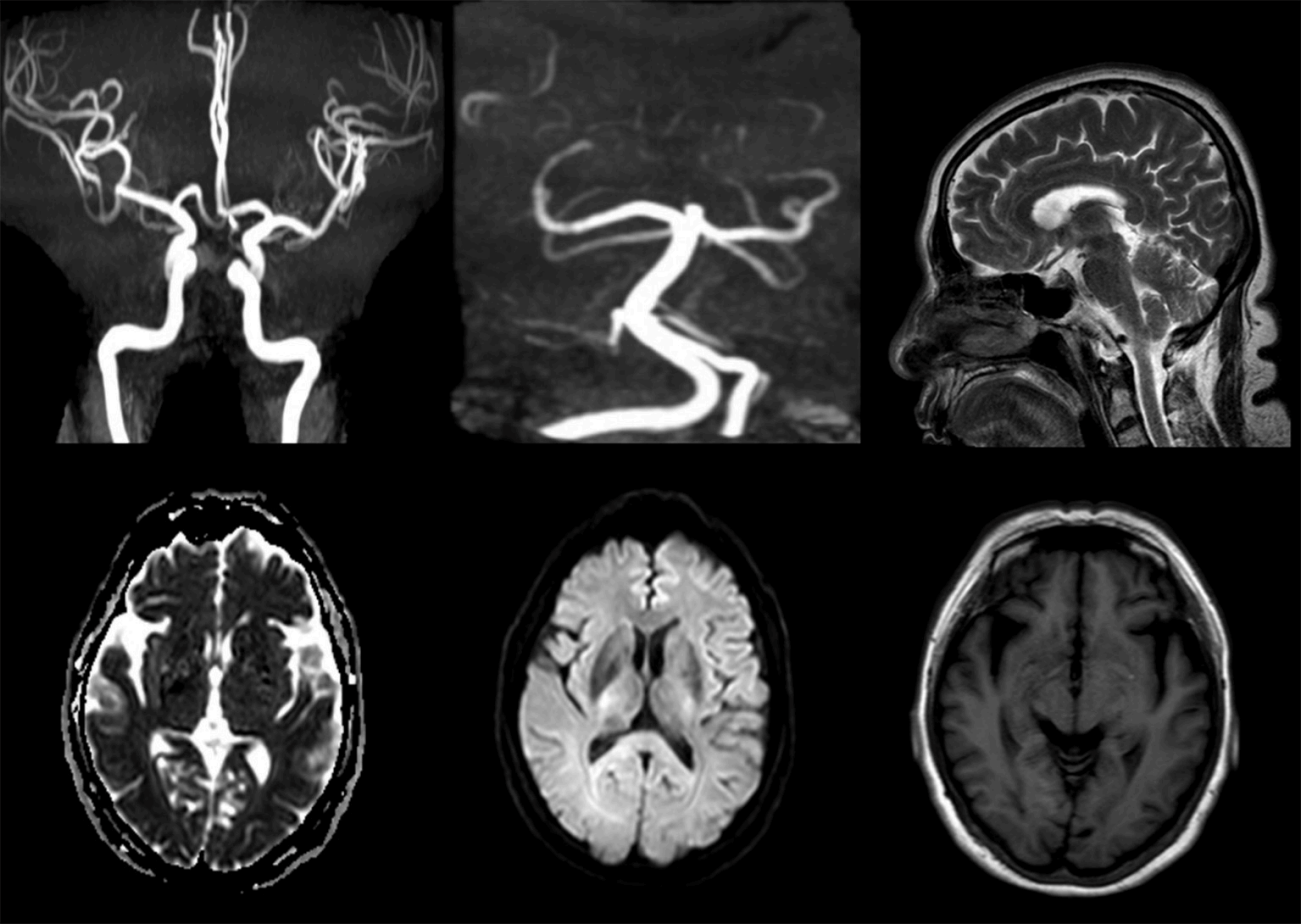
MRA and MRI images of the female patient show no meaningful abnormalities.

A 62-year-old woman (Figure 1, II-3) was admitted to the hospital for recurrent partial headache with weakness of one side and aphasia for about 45 years. In her first attack, the patient suddenly experienced an aura with visual disturbances which she described as increasing scotomata in the bilateral visual field. After a few seconds, the patient developed a serious headache, mainly located on the left side. After a few minutes, she presented a paralysis of the right side and speech difficulties accompanied by dizziness and vomiting. These symptoms resolved after about 2 h. After this initial onset, she had an attack nearly every 4–5 years, and the clinical presentations of her attacks were always similar to the first one. The duration of the aura symptoms and the migraine was typically 1–2 h but sometimes the migraine could last up to 4 days. Sometimes headaches occurred before the hemiplegia and aphasia. Each headache was accompanied by dizziness and vomiting but without loss of consciousness. In most attacks, this patient experienced additionally a flushing of the neck and face and felt that the skin temperature of this affected area was increased, but the temperature was never measured. These symptoms may be related to an extracranial vasodilation when a migraine attack occurred. She did not undergo regular treatment except for simple analgesics as a symptomatic therapy. Recently, her condition aggravated as the frequency of attacks increased from once every 4–5 years to once every 1–2 weeks which had a serious impact on her everyday life. Therefore, during a severe migraine attack, she visited our hospital. We reviewed her family history, and we found that three other subjects, her mother, brother, and nephew, had similar clinical symptoms (Figure 1). Their presentations are as follows:

The proband's mother (Figure 1, I-2) died of uremia at the age of 72. According to her husband and children, she reported typical hemiplegic migraines since an age of 14 years with five attacks per year on average. The aura symptoms were similar to those in the proband, including bilateral visual symptoms (scotomata), speech difficulties, and hemiparesis.

The proband's 55-year-old brother (Figure 1, II-1) had first at the age of 15 headache attacks with nausea, vomiting, visual field defects, and one-sided motor weakness. Usually, these attacks last 5 h. The disease presentation was progressive with age.

The proband's nephew (Figure 1, III-1), a 25-year-old fitness coach, had first headache attacks with visual symptoms (scotomata) and lateralized motor weakness at the age of 13. Each attack lasts about 20 min.

After admission, her neurological examinations were unremarkable and brain magnetic resonance imaging (MRI) and Magnetic Resonance Angiography (MRA) showed no meaningful abnormalities (Figure 2). Thus, the suspected diagnosis was transient ischemic attack (TIA). During her hospitalization, the patient had several migraine attacks that were characterized first by visual symptoms, then aphasia and right limb paralysis 10 min later, and finally severe headaches after 20 min. At that time, the neurological examination revealed: no loss of consciousness, motor aphasia, muscle strength 2 in the right limb, and normal findings in the examination of the residual nervous system. After about 1 h, the symptoms of the aura were relieved, while the headache lasted for about 1 day. However, the symptoms were not relieved after dual antiplatelet aggregation treatment, and transthoracic echocardiography and carotid ultrasound failed to identify any underlying cerebrovascular etiology. After careful consideration of all aspects, she was diagnosed with hemiplegic migraine. So, we conducted a genetic test on the patient and found a heterozygous point mutation (c.4495T>C) in exon 26 of the *SCN1A* gene. This mutation caused amino acid 1499 to change from phenylalanine to leucine (p. Phe1499Leu), which may cause the disease by affecting the SCN1A protein function.

**Figure 3:**
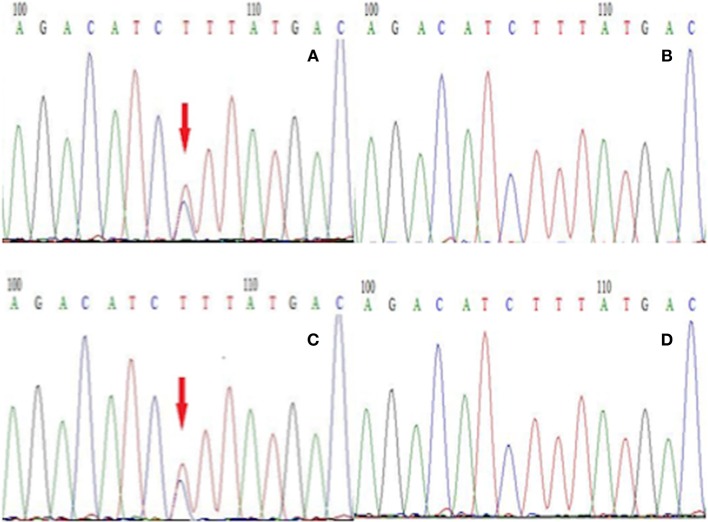
The next-generation sequencing results of exon 26 of the gene SCN1A in members of this family. **(A)** Proband with the T → C mutation, **(B)** the proband's unaffected son without the T → C mutation, **(C)** the proband's brother with the T → C mutation, **(D)** the proband's unaffected granddaughter without the T → C mutation.

To establish the diagnosis, we performed a genetic test on those family members to analyze for the presence of mutations in genes including *CACNA1A, ATP1A2*, and *SCN1A* related to FHM. We only found a gene mutation in *SCN1A*, but this mutation was detected in all affected subjects in this family (Figure 3). Therefore, this patient was diagnosed with FHM3. She was discharged after receiving a health education on migraine attacks, which suggested her staying away from stress, bright lights, sleep disturbances, physical exertion, and alcohol consumption because these have all been reported as trigger factors in FHM (7). Upon being discharged from the hospital, she had intermittently taken flunarizine capsules and rizatriptan benzoate tablets to prevent and control migraine attacks. After 6 months of follow-up, the efficacy of the drug was uncertain, because the frequency of headache attacks was not adequately reduced. After the low efficacy of her medication became clear, we consulted again the literature and consider now a trial with lamotrigine or acetazolamide (8).

## Discussion

Migraine is a common, disabling, multifactorial neurological disorder affecting about 12% of the population, and about 35% of migraineurs experience auras (9). FHM3 is a rare type of migraine with aura which is characterized by focal neurological symptoms such as sensory and motor disturbances. It is notable that all auras can be recoverable after attacks (10). FHM3 may become symptomatic at any age (11), but in this case, it started in all affected family members during adolescence.

Migraine is characterized by a heterogeneous genetic background (10), while FHM is a monogenic autosomal dominant form. Despite numerous studies, the molecular mechanisms of FHM still remain largely unknown but several involved genes have been identified. In this case, the patient presented a mutation in the *SCN1A* gene with the heterozygous nucleotide variation c.4495T>C. This is a missense mutation (p. Phe1499Leu) that may affect the protein function. The *SCN1A* gene encodes for the α1 subunit of the Na_v_1.1 sodium channel including four homologous domains (DI-DIV) with each domain containing six transmembrane segments (6). The Phe1499Leu mutation is located in the intracellular loop between the IIIS6 and IVS1 transmembrane domains (5). The sequence of this region, and more particularly Phe1499, is highly conserved throughout evolution and plays an important role in the function of this protein. In our study, the Phe1499Leu mutation was found in all affected members (Figure 1, II-1, II-3, III-1) and was absent from other unaffected family members (Figure 1) which co-segregates with the FHM phenotype. No mutations of *CACNA1A* and *ATP1A2* gene were found in this family. These results indicate that this mutation (Phe1499Leu) is responsible for the disease. Many mutations in the *SCN1A* gene have been reported in epilepsy, of which c.787C>G and c.3521C>G reportedly co-segregate with FHM and epilepsy (3). However, no epilepsy symptoms were observed in this case, and none of the family members reported any typical signs of epilepsy.

Diagnosis of FHM mainly depends on genetic testing. Early genetic diagnosis for suspected cases can prevent misdiagnosis and reduce overtreatment. This patient was initially wrongly diagnosed with TIA due to the reversibility of the symptoms and the normal imaging findings. Due to the genetic test, this patient was finally diagnosed with FHM. Both TIA and FHM can be described as transient reversible neurological deficit syndromes. A TIA lasts for a short time, generally no more than 24 h. Cerebral vascular examinations and microemboli tests often have positive findings, and dual antiplatelet aggregation therapy is effective. Our case also suggests that the family history is particularly important for an FHM diagnosis. In this case, we could have prevented a misdiagnosis if we had focused earlier on the patient's family history.

It is now recognized that triptans and calcium antagonists are better treatment options for FHM (12). However, the patient's condition has not been significantly improved after the application of the above drugs.

In conclusion, FHM is one of the monogenic autosomal dominant forms of migraine. Therefore, a detailed family history is essential for the diagnosis. Our report is aimed at increasing information about different FHM3 mutations in the available databases and facilitate an understanding of the underlying pathophysiological mechanisms in FHM. This will help to improve the clinical diagnosis of FHM, while a database analysis of normal and pathogenic alleles underlying FHM is a promising route to discover critical molecular targets against which new drugs can be developed.

## Ethics statement

All subjects gave written informed consent to the publication of the information and images related to this case report. As this is a case report without experimental intervention, no formal research ethics approval is required.

## Author contributions

HM wrote the first draft of this manuscript. NS and HZ helped revising the manuscript and collected data. XW, WZ, and MY analyzed and interpreted the data, revised the manuscript, and gave final approval.

### Conflict of interest statement

The authors declare that the research was conducted in the absence of any commercial or financial relationships that could be construed as a potential conflict of interest.
